# Real-time amplitude spectrum area estimation during chest compression from the ECG waveform using a 1D convolutional neural network

**DOI:** 10.3389/fphys.2023.1113524

**Published:** 2023-04-20

**Authors:** Feng Zuo, Chenxi Dai, Liang Wei, Yushun Gong, Changlin Yin, Yongqin Li

**Affiliations:** ^1^ Department of Biomedical Engineering and Imaging Medicine, Army Medical University, Chongqing, China; ^2^ State Key Laboratory of Pathogen and Biosecurity, Beijing Institute of Microbiology and Epidemiology, Beijing, China; ^3^ Department of Intensive Care, Southwest Hospital, Army Medical University, Chongqing, China

**Keywords:** amplitude spectrum area, cardiopulmonary resuscitation, chest compression, convolutional neural network, ventricular fibrillation

## Abstract

**Introduction:** Amplitude spectrum area (AMSA) is a well-established measure than can predict defibrillation outcome and guiding individualized resuscitation of ventricular fibrillation (VF) patients. However, accurate AMSA can only be calculated during cardiopulmonary resuscitation (CPR) pause due to artifacts produced by chest compression (CC). In this study, we developed a real-time AMSA estimation algorithm using a convolutional neural network (CNN).

**Methods:** Data were collected from 698 patients, and the AMSA calculated from the uncorrupted signals served as the true value for both uncorrupted and the adjacent corrupted signals. An architecture consisting of a 6-layer 1D CNN and 3 fully connected layers was developed for AMSA estimation. A 5-fold cross-validation procedure was used to train, validate and optimize the algorithm. An independent testing set comprised of simulated data, real-life CC corrupted data, and preshock data was used to evaluate the performance.

**Results:** The mean absolute error, root mean square error, percentage root mean square difference and correlation coefficient were 2.182/1.951 mVHz, 2.957/2.574 mVHz, 22.887/28.649% and 0.804/0.888 for simulated and real-life testing data, respectively. The area under the receiver operating characteristic curve regarding predicting defibrillation success was 0.835, which was comparable to that of 0.849 using the true value of the AMSA.

**Conclusions:** AMSA can be accurately estimated during uninterrupted CPR using the proposed method.

## 1 Introduction

Out-of-hospital cardiac arrest (OHCA) is a major public health issue and the most common cause of death worldwide (Kiguchi et al., 2020). Defined as a disorganized electrical activity without the presence of distinguishable QRS complexes, ventricular fibrillation (VF) is the most common etiology in patients suffering from OHCA. Although patients with VF as an initial rhythm were more likely to be successfully resuscitated than with other rhythms, the less than 30% survival rate remains unsatisfactory (Rajan et al., 2017). Survival from OHCA depends on a complex system working together to secure the best outcome, and early defibrillation with concurrent high-quality cardiopulmonary resuscitation (CPR) is the most important life-saving intervention for VF (Koike et al., 2011). To provide a general treatment strategy, the latest guidelines for CPR and emergency cardiovascular care recommend the initiation of high-quality CPR, delivery of an electrical shock as soon as a defibrillator is available and every 2 min thereafter if VF persists (Panchal et al., 2020).

With the deepening understanding of the physiology of cardiac arrest and resuscitation, it is increasingly clear that not all patients in VF benefit from being treated with the same intervention. The development of new technologies also enables unprecedented ability to personalize resuscitation according to the interval after onset of the VF, the effectiveness of CPR and the consequent myocardial metabolic state (Chalkias et al., 2019). Electrocardiogram (ECG) waveforms, which are routinely collected by the automated external defibrillators (AEDs), have been extensively investigated to identifying VFs and predicting defibrillation outcomes (Bessen et al., 2021). Based on the observation that the characteristics of VF signals reflect the pathophysiological and metabolic status of the fibrillating heart, a variety of measures have been developed to characterize the underlying organization of the myocardial electrical activity and with the ultimate goal of guiding CPR (Coult et al., 2019). Amplitude spectrum area (AMSA), as a well-established measure to predict defibrillation outcome, has been shown to be correlated with coronary perfusion pressure, reflect the energy state of the myocardium, and reveal whether myocardial perfusion is improved (Reynolds et al., 2012). Clinical studies have confirmed that both preshock AMSA and relative changes in the AMSA during CPR are associated with shock success (Indik et al., 2014; Schoene et al., 2014; Ristagno et al., 2015). Therefore, real-time monitoring of AMSA may serve as a strategy for quality control of CPR and for individualization of resuscitation (Babini et al., 2021).

Unfortunately, real-time monitoring of AMSA remains unachievable with present algorithms. Because the mechanical activity of chest compression (CC) induces artifacts in the ECG, reliable AMSA calculation can only be achieved during CC pauses. The life-saving benefit of CPR will be markedly compromised if CC is repetitively interrupted to calculate AMSA during resuscitation efforts (Shimizu et al., 2021). Therefore, it is a major challenge for accurate AMSA assessment during uninterrupted CPR to complete the goal of patient specific, time-sensitive and physiology-directed strategy of personalized resuscitation.

To perform reliable and accurate ECG waveform analysis without interrupting CPR, a number of signal processing solutions have been proposed to remove CC artifacts in the past two decades (Gong et al., 2013). One solution is to suppress artifacts using only the ECG waveform, such as the Kalman filter (Ruiz de Gauna et al., 2008), independent component analysis (Granegger et al., 2011), coherent line removal algorithm (Amann et al., 2010), empirical mode decomposition (Lo et al., 2013) and condition-based filtering algorithm (Hajeb-Mohammadalipour et al., 2021). Although artifacts can be strongly suppressed, and the signal-to-noise ratio (SNR) is markedly improved, the specificity for VF detection is insignificantly improved using these methods. The other solution is to remove the artifacts using additional CPR-related reference waveforms, including Gabor multiplier (Werther et al., 2009), Wiener filter (Aase et al., 2000), recursive adaptive matching pursuit algorithm (Husøy et al., 2002), adaptive filtering based on the least mean square (LMS) algorithm (Irusta et al., 2009; Gong et al., 2017) and variable-frequency notch filter (Coult et al., 2021). CPR artifacts are first modeled by a reference waveform, such as compression depth, transthoracic impedance, and compression force, and are subsequently subtracted from the corrupt waveform (Ruiz de Gauna et al., 2014). Although the time-frequency variability of the artifacts can be reconstructed to a certain extent, additional equipment is required to obtain these references, and they are not available in all existing AEDs.

Recently, powerful tools developed for machine learning have been successfully applied in the field of biomedical signal processing by end-to-end architectures of deep neural networks (Li et al., 2021). A currently popular tool is the convolutional neural network (CNN), which is a hierarchical neural network model with alternating convolutional and subsampling layers, followed by a fully connected layer that is identical to a multilayer perceptron. The biggest advantage of CNN is that it convolves the learned features with the input data without manually extracting features. Inspired by the feature learning capacity of CNN for image classification, CNNs have also been shown to be able to classify non-image time series and waveform data. Specifically, several attempts have been made in ECG waveform analysis, such as signal denoising (Fotiadou et al., 2020), QRS detection (Zahid et al., 2022), heartbeat classification (Kiranyaz et al., 2016), arrhythmia detection (Hannun et al., 2019) and defibrillation success prediction (Ivanović et al., 2020). Although these studies demonstrated that CNNs have a wide application prospect in ECG waveform analysis, they focused mainly on the problem of signal classification and/or tested on clinical ECG records with low-level motion artifacts. Therefore, whether CNN can be used for reliable VF signal analysis during CPR when the ECG waveform is severely corrupted with a high level of artifacts remains unsolved.

In this paper, we propose a novel AMSA estimation algorithm that can provide continuous guidance for personalizing CPR in realtime during resuscitation efforts using a 1D CNN. To accomplish this, an architecture consisting of 1D CNN blocks and fully connected layers is used for feature extraction and AMSA estimation. The proposed approach can estimate AMSA from CC-corrupted VF signals without reference, thus, eliminating the need for artifact filtering, feature extraction and postprocessing.

## 2 Materials

### 2.1 Data collection and extraction

This study was approved by the Medical Ethics Committee of the Army Medical University (2020-002-02). Written informed consent was waived due to the study’s retrospective nature. The data used in this study were recorded by defibrillators/AEDs from 728 adult patients who experienced non-traumatic OHCA and CPR between February 2010 and December 2020. The presenting cardiac rhythm was VF in 698 cases and asystole in 30 cases. In addition to the ECG waveform acquired from defibrillation pads, an additional CPR-related reference waveform was also simultaneously recorded in this dataset. In 474 cases, ECG and compression depth waveforms were recorded through two standard adhesive adult defibrillation/pacing pads that integrated an accelerometer-based CPR feedback at a sample rate of 250 Hz using ZOLL defibrillators/AEDs (E/R series and AED pro, ZOLL Medical Corporation, Chelmsford, MA, United States). In the other 254 cases, the ECG waveform was recorded at a sample rate of 125 Hz and the transthoracic impedance waveform was recorded from the same defibrillation pads at a sample rate of 60 Hz using Physio-Control defibrillators/AEDs (LIFEPAK series, Physio-Control, Redmond, WA, United States). The waveforms were resampled to 250 Hz for compatibility and analyzed using MATLAB (version R2020a, The MathWorks, Inc., Natick, MA, United States) software.

**FIGURE 1:**
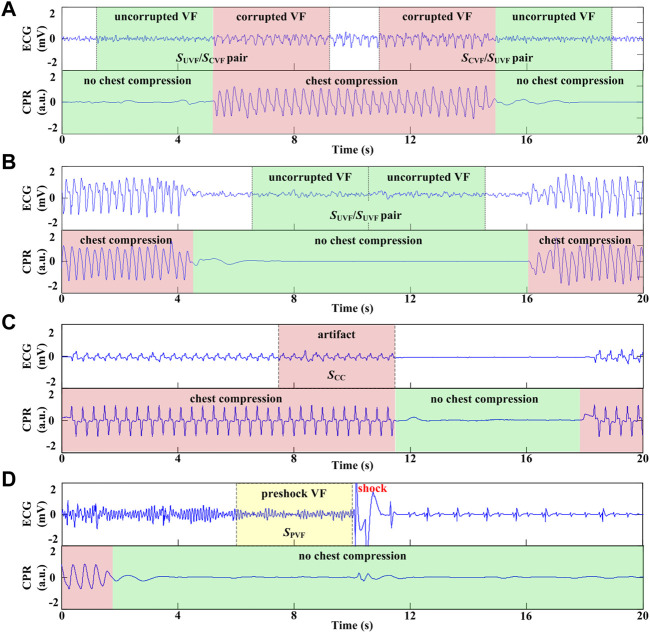
Process and different types of ECG segment extraction. **(A)** 8-s VF signals including 4s with CC and adjacent 4s without CC. **(B)** 8-s VF signals without CC. **(C)** 4-s ECG segment during CC when the underlying rhythm was asystole. **(D)** 4-s VF signals without CC prior to defibrillation shock.

For each case, the cardiac rhythm and presence/absence of CCs were annotated by two experienced medical doctors. The symbols and acronyms used in the current study are listed in Table 1. As shown in Figures 1, 4 types of ECG segments were extracted from the recordings.

(1) *S*_UVF_/*S*_CVF_ segment pairs: A 4-s uncorrupted VF signal without CC followed by an adjacent 4-s corrupted VF signal with CC, or *vice versa* (*S*_CVF_/*S*_UVF_ pairs) (Figure 1A).
(2) *S*_UVF_/*S*_UVF_ segment pairs: 2 consecutive 4-s uncorrupted VF signals without CC (Figure 1B).
(3) Pure artifact segment *S*_CC_: a 4-s ECG signal during CC when the underlying rhythm was asystole and without cardiac electrical activity in 30 cases (Figure 1C).
(4) Preshock segment *S*_PVF_: a 4-s uncorrupted VF signal prior to a defibrillation shock without CC with a presenting cardiac rhythm of VF in the 138 cases from testing set (Figure 1D).

**TABLE 1:** symbols and acronyms used in the current study.

Symbol	Acronym
S_UVF_	4-s uncorrupted VF signal without CC
S_AVF_	adjacent 4-s uncorrupted VF signal of a S_UVF_ without CC
S_CVF_	4-s corrupted VF signal with CC
S_CC_	4-s ECG signal during CC when the underlying rhythm is asystole
S_SVF_	4-s simulated corrupted VF signal with known AMSA and SNR
S_PVF_	4-s uncorrupted VF signal prior to a defibrillation shock
P_UVF_	power of a S_UVF_
P_CC_	power of a S_CC_
AMSA_FFT_AVF	AMSA value of a S_AVF_ calculated using FFT method
AMSA_FFT_UVF	AMSA value of a S_UVF_ calculated using FFT method
AMSA_FFT_SVF	AMSA value of a S_SVF_ calculated using FFT method
AMSA_ADF_SVF	AMSA value of a S_SVF_ calculated using ADF method
AMSA_FFT_CVF	AMSA value of a S_CVF_ calculated using FFT method
AMSA_ADF_CVF	AMSA value of a S_CVF_ calculated using ADF method
AMSA_CNN_CVF	AMSA value of a S_CVF_ calculated using CNN method
AMSA_FFT_PVF	AMSA value of a S_PVF_ calculated using FFT method
AMSA_CNN_PVF	AMSA value of a S_PVF_ calculated using CNN method

VF, ventricular fibrillation; CC, chest compression; AMSA, amplitude spectrum area; SNR, signal-to-noise ratio, FFT, fast Fourier transformation; ADF, adaptive filtering with the least mean square algorithm; CNN, convolutional neural network.

### 2.2 Dataset construction and data labeling

Two datasets (i.e., the derivation set and the testing set) were constructed using the extracted ECG segments. The derivation set used to develop the algorithm consisted of the *S*_UVF_/*S*_CVF_ and *S*_CVF_/*S*_UVF_ pairs from 560 cases. For each segment pair, the AMSA calculated from the uncorrupted VF signal *S*_UVF_ based on fast Fourier transformation (FFT) was labeled as the true value of both segments. The derivation set was then used to create to a training set (448 cases) and a validation set (112 cases) to validate and optimize the model. The testing set (138 cases) used to evaluate the algorithm comprised 3 parts.

(1) Simulated data. The uncorrupted VF signals originated from patients in the testing set but were extracted from different time periods. The simulated corrupted VF signal *S*_SVF_ was constructed by randomly adding a scaled pure artifact signal *S*_CC_ to one of the uncorrupted VF signal *S*_UVF_/*S*_UVF_ pairs at 4 SNR levels (3 dB, 0 dB, −3 dB and −6 dB) (Aramendi et al., 2007).

$$S_{SVF}\left(i\right)=S_{UVF}\left(i\right)+\sqrt{P_{UVF}/P_{CC}10^{\left(SNR/10\right)}}\cdot S_{CC}\left(i\right)$$ (1)

where *P*_UVF_ is the power of *S*_UVF_ and *P*_CC_ is the power of *S*_CC_. For each *S*_SVF_, the AMSA calculated from the original *S*_UVF_ based on FFT was labeled as the true value.

(2) Real-life data. Similar to the derivation set, the *S*_UVF_/*S*_CVF_ and *S*_CVF_/*S*_UVF_ pairs from an additional 138 cases served as testing data, while AMSA calculated from the adjacent *S*_UVF_ based on FFT was labeled as true value.
(3) Preshock data. The preshock VF signal *S*_PVF_ with annotated cardiac rhythm after each defibrillation shock constituted the testing set, and the AMSA calculated by the FFT method was labeled its true value. The FFT-based AMSA is calculated as the sum of the products of individual frequencies and their amplitudes converted from the time to the frequency domain by FFT using a Tukey window (Li et al., 2013):

$$AMSA\_FFT=\sum_{2}^{48}S\left(f\right)fdf$$ (2)

where *S*(*f*) and *f* are the spectrum and frequency of a signal *s*(*n*). The lower and upper limits of *f* for integral summation are 2 Hz and 48 Hz, respectively.

### 2.3 Rhythm annotation

VF was defined as a disorganized, chaotic rhythm with a median peak-to-peak amplitude >0.1 mV, while asystole was defined as an isoelectric ECG with a peak-to-peak amplitude <0.1 mV. A defibrillation shock was regarded as successful when VF was converted to an organized rhythm with a heart rate greater than 40 beats/min and sustained for a period greater than 30 s (Jin et al., 2017).

## 3 Methods

**FIGURE 2:**
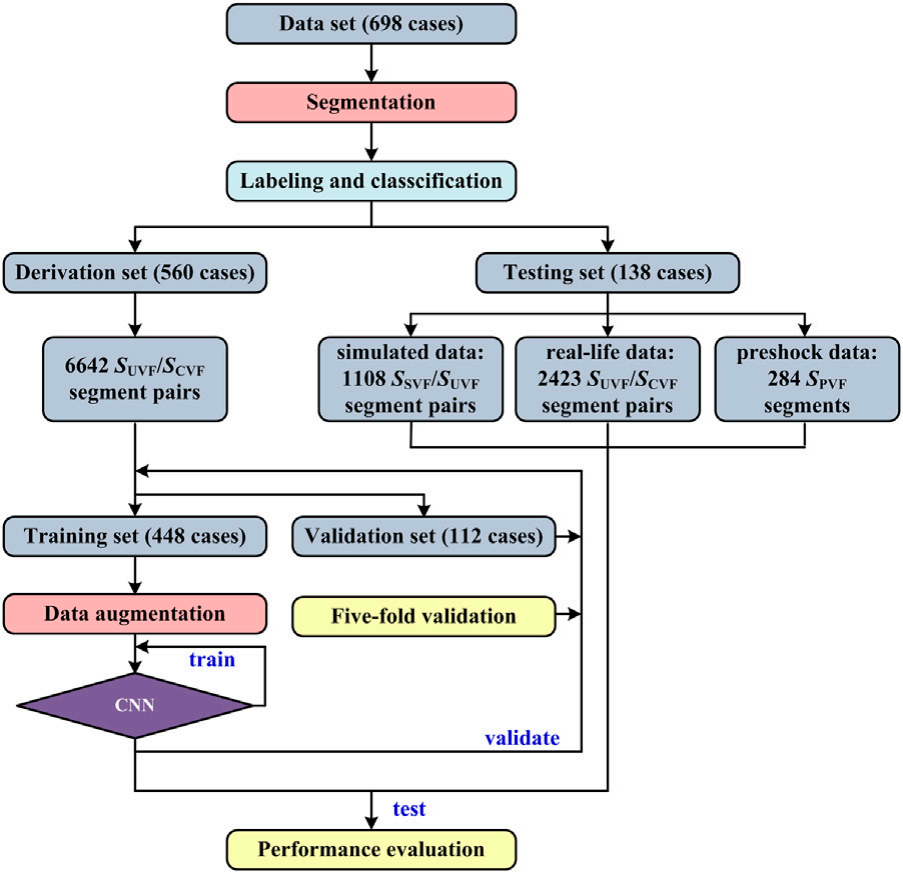
Flowchart of this study.

The flowchart of this study is shown in Figure 2. First, the VF signals were preprocessed, labeled and distributed to different datasets. Second, the model was trained by the augmented training data and optimized by the validation data. Finally, the performance of the developed algorithm was evaluated using the testing data and compared with traditional adaptive filtering and FFT-based techniques. There was no crossover between subjects in the training, validation and testing sets.

### 3.1 Data preprocessing

The 4-s VF signals were filtered using a second-order high-pass filter with a cut off frequency at 0.5 Hz to remove baseline drift. Two-channel signals were then transformed by downsampling and differential operation to enhance the frequency change of VF and to improve the accuracy of estimation:

$$S_{d1}\left(i\right)=S\left(2i+1\right)-S\left(2i-1\right)$$ (3)

$$S_{d2}\left(i\right)=S\left(2i+2\right)-S\left(2i\right)$$ (4)

where *S* is the filtered VF signal; [*S*_d1_; *S*_d2_] is the reconstructed signal; *i* = 1, 2, … , *L*-1; and *L* is the length of *S*.

### 3.2 CNN architecture

**FIGURE 3:**
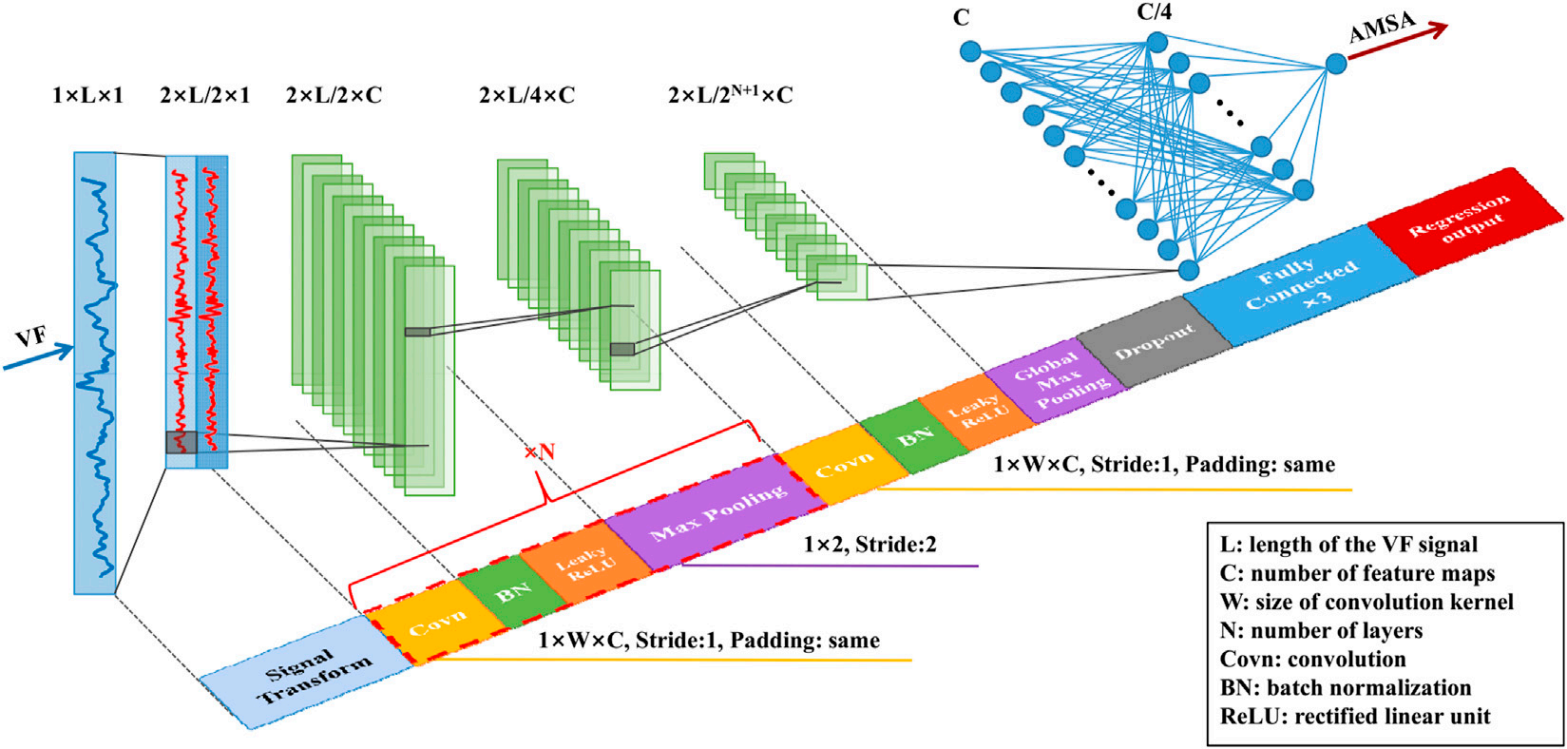
Architecture of the proposed algorithm.

The architecture of the proposed model was adapted from the typical 1D CNNs that have been successfully applied for ECG time series (Hannun et al., 2019; Ivanović et al., 2020; Li et al., 2021). As shown in Figure 3, the preprocessed signals were fed into the *N*+1 blocks of the CNN feature extractor first. Each feature extraction block was composed of 4 stages: convolution, batch normalization (BN), leaky rectified linear unit (ReLU) activation and pooling. The size of the convolution kernel was 1 × *W* × *C* with a stride of 1 and “same” padding to ensure that the output size was the same as the input size. BN layers were added after each convolution layer to stabilize training and fast convergence. ReLU activation was introduced because it could speed up learning and mitigate the vanishing gradient problem. Max-pooling was used for the first *N* blocks to subsample the feature maps with a pool kernel of 1 × 2 and a stride of 2. Additionally, global max-pooling and dropout techniques were used in the last convolutional block to improve the generalization capability. Then, the output of the feature extractor was flattened and fed into three fully connected layers. Finally, an AMSA value was output using a general regression function.

The hyperparameters including the number of channels (*C*, ranging from 22 to 26 with a scale factor of 2), the size of the convolution kernel (*W*, ranging from 3 to 11 with a step of 2) and the number of layers (*N*, ranging from 1 to 7 with a step of 1), were optimized by the grid search method using the training set.

### 3.3 Data augmentation

Considering the relatively limited sample size of the training set, a data augmentation technique was applied to avoid overfitting and improve the robustness and generalization ability in training the model. Combinations of three types of operations, including taking opposite numbers, flipping horizontally and flipping vertically, were applied to the reconstructed signals as follows:

$${\left[S_{d1};S_{d2}\right]}_{1}=\left[-S_{d1}\left(1\right),\cdots ,-S_{d1}\left(L/2\right);-S_{d2}\left(1\right),\cdots ,-S_{d2}\left(L/2\right)\right]$$ (5)

$${\left[S_{d1};S_{d2}\right]}_{2}=\left[S_{d1}\left(L/2\right),\cdots ,S_{d1}\left(1\right);S_{d2}\left(L/2\right),\cdots ,S_{d2}\left(1\right)\right]$$ (6)

$${\left[S_{d1};S_{d2}\right]}_{3}=\left[S_{d2}\left(1\right),\cdots ,S_{d2}\left(L/2\right);S_{d1}\left(1\right),\cdots ,S_{d1}\left(L/2\right)\right]$$ (7)

$${\left[S_{d1};S_{d2}\right]}_{4}=\left[-S_{d1}\left(L/2\right),\cdots ,-S_{d1}\left(1\right);-S_{d2}\left(L/2\right),\cdots ,-S_{d2}\left(1\right)\right]$$ (8)

$${\left[S_{d1};S_{d2}\right]}_{5}=\left[-S_{d2}\left(1\right),\cdots ,-S_{d2}\left(L/2\right);-S_{d1}\left(1\right),\cdots ,-S_{d1}\left(L/2\right)\right]$$ (9)

$${\left[S_{d1};S_{d2}\right]}_{6}=\left[S_{d2}\left(L/2\right),\cdots ,S_{d2}\left(1\right);S_{d1}\left(L/2\right),\cdots ,S_{d1}\left(1\right)\right]$$ (10)

$${\left[S_{d1};S_{d2}\right]}_{7}=\left[-S_{d2}\left(L/2\right),\cdots ,-S_{d2}\left(1\right);-S_{d1}\left(L/2\right),\cdots ,-S_{d1}\left(1\right)\right]$$ (11)

### 3.4 Model training

The model was developed using the derivation set with a fivefold cross-validation method because a decreased validation sample size may decrease the resolution of validation. In each iteration, 448 cases (80%) were randomized to the training set, and the additional 112 cases (20%) were randomized to the validation set. The model was trained by optimizing the mean squared error objective function using the Adam optimizer with the default parameters and a learning rate of 10^−3^. The parameters of the model were initialized randomly in the range of [−0.1, 0.1]. Data in the training set were randomly shuffled and divided into mini-batches with a size of 1,024 to speed up the convergence speed. Training was performed out from scratch in 50 epochs by initializing the weights of the convolutional layers using the Xavier normal initializer. After training the network, the entire validation set was propagated through the network to evaluate the performance. Overall performances were obtained by averaging the performance metrics recorded in each fold of the cross-validation. The parameters of the model were finally trained using the entire derivation set after the hyperparameters were determined.

### 3.5 Comparison methods

The AMSA values calculated directly from the corrupted VF signals using the FFT-based method (AMSA_FFT) and calculated after adaptive filtering with the LMS algorithm (AMSA_ADF) were used to compare with the performance of the proposed method.

The cycle length of each CC was identified from the reference waveform. The instantaneous rate of each compression was then determined by the inverse of the compression cycle length. The estimation of *S*_CC_ is adaptively computed and subtracted from the input signal *S*_CVF_ to produce an estimated VF signal *S*_EVF_. The model of CPR artifacts was:

$$\left.{\hat{S}}_{CC}\left(i\right)=\sum_{k=1}^{L}\left(a_{k}\left(i\right)\cos\left(2\pi kf_{0}\left(n\right)i/f_{s}\right)+\right.b_{k}\left(i\right)\sin\left(2\pi kf_{0}\left(n\right)i/f_{s}\right)\right))$$ (12)

where *f*_0_ (*n*) is the time-varying frequency of the *n*th compression calculated by the inverse of the cycle length; *a*_*k*_(*i*) and *b*_*k*_ (*i)* are the magnitudes of the sinusoidal harmonics of the filter; *f*_*s*_ is the sampling rate; and *k* is the order of harmonics. The LMS method was applied for estimating and updating *a*_*k*_ (*i*) and *b*_*k*_ (*i*) using increments proportional to the squared error and the step-size with the criteria to minimize the error between *S*_CVF_ and ${\hat{S}}_{CC}$ (Lo et al., 2013). The estimated VF signal was then described by follows:

$$S_{EVF}\left(i\right)=S_{CVF}\left(i\right)-{\hat{S}}_{CC}\left(i\right)$$ (13)

AMSA_FFT and AMSA_ADF were calculated using Equation 2 to *S*_CVF_ and *S*_EVF_ individually.

### 3.6 Performance evaluation and statistical analysis

AMSA values estimated with the proposed method (AMSA_CNN) were evaluated and compared with AMSA_FFT and AMSA_ADF using the testing set. The measures used to quantify the performance were correlation coefficient (*r*), mean absolute error (MAE), root mean square error (RMSE), percentage root mean square difference (PRD) and area under the receiver operating characteristic curve (AUC). MAE, RMSE and PRD were calculated using the following equations:

$$MAE=\frac{1}{N}\sum_{i=1}^{N}\left|y_{i}-{\hat{y}}_{i}\right|$$ (14)

$$RMSE=\sqrt{\frac{1}{N}\sum_{i=1}^{N}{\left(y_{i}-{\hat{y}}_{i}\right)}^{2}}$$ (15)

$$PRD=\sqrt{\sum_{i=1}^{N}{\left(y_{i}-{\hat{y}}_{i}\right)}^{2}/\sum_{i=1}^{N}y_{i}^{2}}\times 100\%$$ (16)

where *N* is the total number of segments, and *y*_i_ and ${\hat{y}}_{i}$ are the estimated and true values of the AMSA of segment *i,* respectively.

Data are reported as medians [interquartile ranges] and were compared with the Mann-Whitney *U* test because they were not normally distributed. *r* was investigated by Spearman correlation analysis. The agreement of AMSA between estimated and true values was examined by a Bland-Altman mean-difference plot. The area under the receiver operating characteristic curve (AUC) was compared using the Hanley and McNeil method. Two-sided *p* values of 0.05 were considered statistically significant, and all analyses were conducted with SPSS (version 22, IBM Corp, Armonk, NY, United States).

## 4 Results

A total of 6642 *S*_UVF_/*S*_CVF_ and *S*_CVF_/*S*_UVF_ segment pairs were extracted in the derivation set. The segment pairs randomized to training and validation sets in the five-fold validation iteration are 5,372/1,270, 5,435/1,207, 5,175/1,467, 5,260/1,382 and 5,318/1,324, respectively. Additionally, 1,108 *S*_SVF_/*S*_UVF_ segment pairs, 2,423 *S*_UVF_/*S*_CVF_ or *S*_CVF_/*S*_UVF_ segment pairs and 284 *S*_PVF_ segments (98 successful and 186 unsuccessful shocks) were extracted in the testing set. The optimal hyperparameters of the model were *W* = 11, *N* = 5, and *C* = 32.

### 4.1 Results on simulated data

The AMSA values were 11.573 [5.752] mVHz and 11.460 [5.459] mVHz (*p* = 0.415) for the *S*_UVF_/*S*_UVF_ segment pairs, and the MAE was 1.324 [1.292] mVHz when the AMSA was estimated from the adjacent 4-s segment *S*_AVF_. For the simulated segment *S*_SVF_, AMSA_FFT was significantly higher [17.337 (11.003) mVHz, *p* < 0.001], AMSA_CNN was relatively lower [10.624 (4.308) mVHz, *p* < 0.001], and AMSA_ADF had no significant difference [11.131 (6.746) mVHz, *p* = 0.052] compared to its true value [11.460 (5.459) mVHz].

**FIGURE 4:**
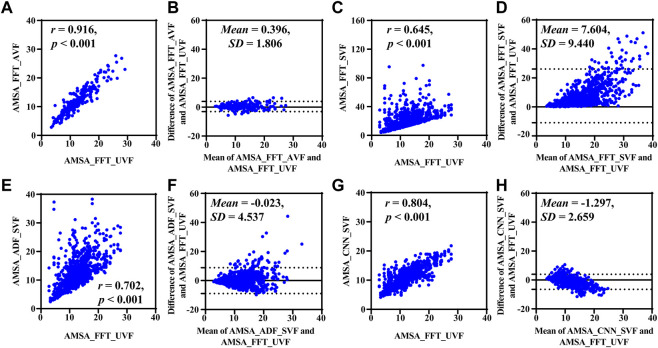
Performance results on simulated data. **(A)** Relationship and **(B)** difference between AMSA_FFT of adjacent VF and true value. **(C)** Relationship and **(D)** difference between the AMSA_FFT of the corrupted VF and true value. **(E)** Relationship and **(F)** difference between AMSA_ADF of corrupted VF and true value. **(G)** Relationship and **(H)** difference between the AMSA_CNN of the corrupted VF and true value.

Results base on simulated data are shown in Figure 4 and Table 2. Compared with the true value, the AMSA calculated from *S*_AVF_ had the highest *r* and lowest MAE, RMSE and PRD values. In contrast, AMSA_FFT was markedly biased in the corrupted signals with the lowest *r* and highest MAE, RMSE and PRD values due to the additive artifacts. Although both AMSA_ADF and AMSA_CNN could efficiently mitigate the bias, AMSA_CNN achieved better performance with higher *r* and lower MAE, RMSE and PRD compared with those of AMSA_ADF.

**TABLE 2:** Performance of different AMSA estimation method using simulation data.

Method	MAE (mVHz)	RMSE (mVHz)	PRD (%)
AMSA_FFT_SVF	7.604 [9.440]	12.119	93.797
AMSA_FFT_UVF	1.319 [1.293] *	1.839	14.288
AMSA_ADF_SVF	3.116 [3.297] * †	4.535	35.100
AMSA_CNN_SVF	2.182 [1.997] *††‡	2.957	22.887

MAE, mean absolute error; RMSE, the root mean square error; PRD, the percentage root mean square difference. **, † and ‡: *p* < 0.05 compared with AMSA_FFT_SVF, AMSA_FFT_UVF, and AMSA_ADF_SVF.

### 4.2 Results on real-life data

**FIGURE 5:**
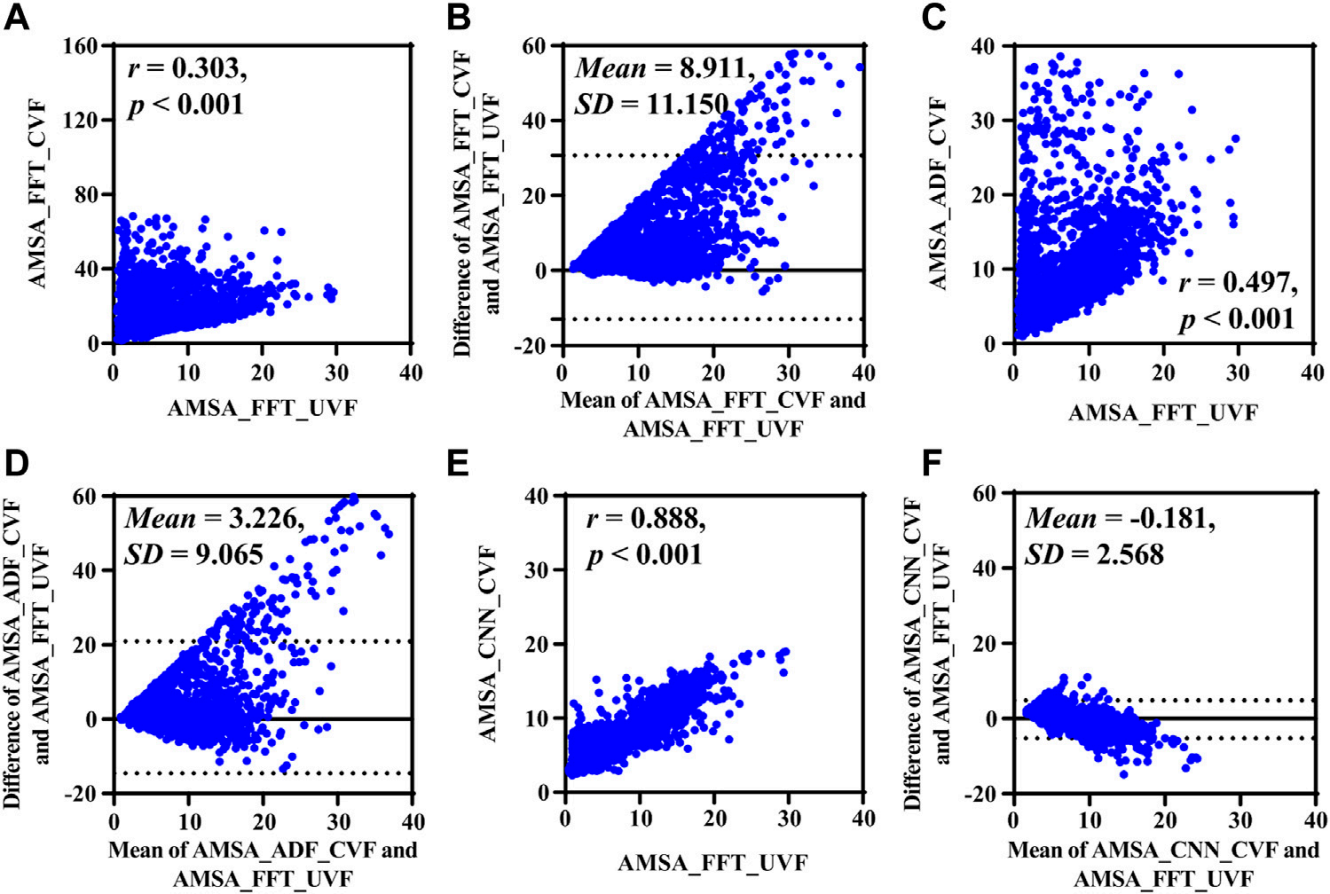
Performance results on real-life data. **(A)** Relationship and **(B)** difference between AMSA_FFT of corrupted and adjacent uncorrupted VFs. **(C)** Relationship and **(D)** difference between AMSA_ADF of the corrupted VF and AMSA_FFT of the adjacent uncorrupted VF. **(E)** Relationship and **(F)** difference between the AMSA_CNN of the corrupted VF and AMSA_FFT of the adjacent uncorrupted VF.

Results based on real-life data S_CVF_, whose true value was determined by the adjacent segment without CC *S*_UVF_, are listed in Figure 5 and Table 3. Both AMSA_FFT [13.253 (10.571) mVHz, *p* < 0.001] and AMSA_ADF [8.517 (7.485) mVHz, *p* < 0.001] were significantly higher, while AMSA_CNN [7.013 (4.323) mVHz, *p* = 0.405] was comparable to the estimated AMSA true value of 7.303 (7.350) mVHz.

**TABLE 3:** Performance of different AMSA estimation method using test data.

Method	MAE (mVHz)	RMSE (mVHz)	PRD (%)
AMSA_FFT_CVF	9.016 [11.059]	14.267	158.778
AMSA_ADF_CVF	5.002 [8.226] *	9.626	107.123
AMSA_CNN_CVF	1.951 [1.680] * ‡	2.574	28.649

MAE, mean absolute error; RMSE, the root mean square error; PRD, the percentage root mean square difference. * and ‡: *p* < 0.05 compared with AMSA_FFT_CVF, and AMSA_ADF_CVF.

Consistent with the results on simulated data, AMSA_FFT was markedly biased in corrupted signals, and the bias could not be efficiently mitigated by AMSA_ADF, as shown by its lower *r* and higher RMSE and PRD. AMSA_CNN, however, achieved better performance than AMSA_ADF, with relatively higher *r* and lower MAE, RMSE and PRD values.

The Bland-Altman plots showed a bias between the estimated AMSA and its real values. All methods overestimated the AMSA value during CC and the absolute error increases as the true AMSA value increases.

### 4.3 Results on preshock data

**FIGURE 6:**
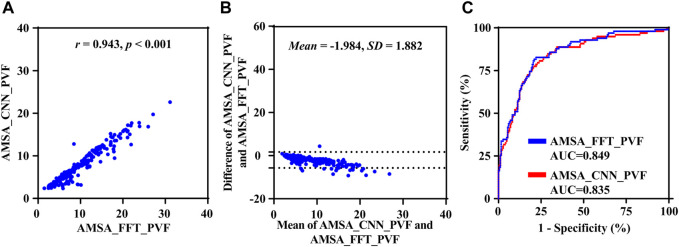
Performance results of the proposed method on preshock data. **(A)** Relationship, **(B)** difference and **(C)** receiver operating characteristic curve between AMSA_CNN and AMSA_FFT.

Results based on the uncorrupted preshock data *S*_PVF_ are shown in Figure 6. The *r*, MAE, RMSE and PRD values between AMSA_CCN and AMSA_FFT were 0.943, 2.047 mV Hz, 2.738 mV Hz and 25.557%, respectively. AMSA was 7.701 (6.700) mVHz for AMSA_CCN and 6.168 (4.840) mVHz for AMSA_FFT (*p* < 0.001). Although the difference between AMSA_CCN and AMSA_FFT was relatively higher than that of the real-life data (−1.984 ± 1.882 vs. −0.181 ± 2.568, *p* < 0.001), the AUC for the prediction of defibrillation success was comparable between AMSA_CCN and AMSA_FFT (0.835 vs. 0.849, *p* = 0.120).

### 4.4 Example of AMSA monitoring during CPR

**FIGURE 7:**
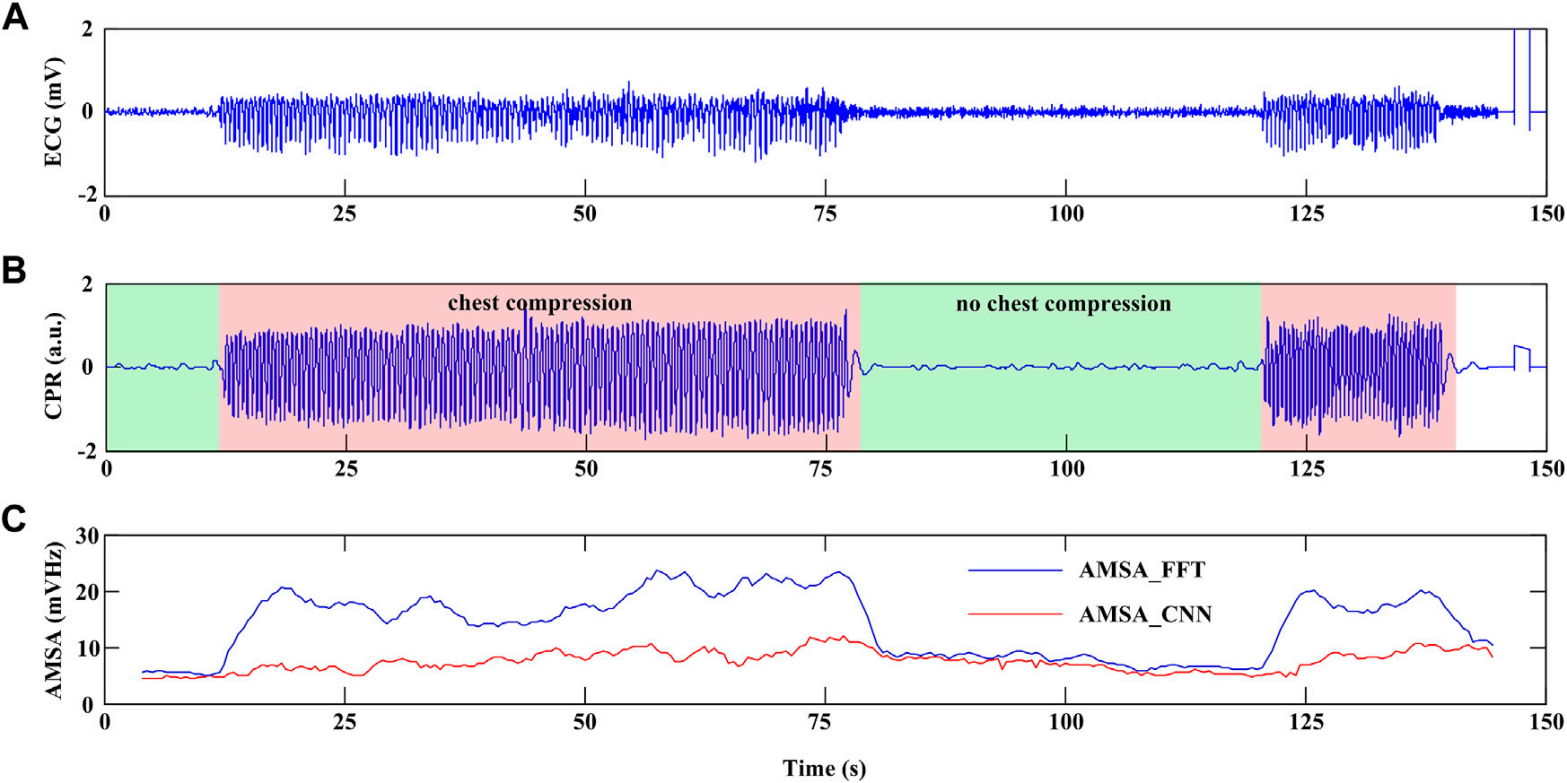
Example of continuous AMSA monitoring during CPR. **(A)** ECG waveform. **(B)** CPR reference signal. **(C)** AMSA_FFT and AMSA_CNN values.

Figure 7 shows an example of continuous AMSA monitoring with a time interval of 0.5 s during CPR using the proposed method. In this case, CC was initiated at 12-s and 120-s in the recording and lasted for 66 s and 20 s, respectively. An interruption of 42 s was observed between two rounds of CC. AMSA_FFT was overestimated during CC and returned to normal when CC was interrupted. However, AMSA_CNN was sustained in a relatively stable state during both interrupted and uninterrupted CCs. Therefore, the effectiveness of CC therefore can be monitored by the absolute AMSA value and its relative change during CPR. In this example, AMSA was increased from 4.861 mV Hz to 11.983 mV Hz during the first round CC but decreased to 5.109 mV Hz after the interruption and improved to 10.312 mV Hz after the second round of CC.

## 5 Discussion

Given the importance of continuous AMSA evaluation during uninterrupted CPR for quality control and personalized defibrillation, this study introduces a solution for reliable AMSA estimation from VF signals using the 1D CNN model. Compared with the traditional AMSA calculation method combining adaptive filtering and FFT techniques, the proposed algorithm can reliably estimate AMSA from VF signals. Also, the proposed method is validated to preserve the predictive performance of defibrillation success.

Analyzing ECG waveforms during uninterrupted CPR remains a major challenge because signals are always contain artifacts. An earlier study indicated that CC-produced artifacts are an additive noise predominantly generated from electrode motion and thoracic muscle contraction (Fitzgibbon et al., 2002). When the underlying cardiac rhythm of a patient is asystole, the ECG waveform recorded during CPR can be regarded as pure artifacts produced by CC (Gong et al., 2013). In the time domain, the artifact features a relatively high amplitude and an nearly periodic waveform. In the frequency domain, the energy content is concentrated around the harmonics of the fundamental frequency being that of the CC with a bandwidth of approximately 0-20 Hz. Because the energy of VF signals lies in the frequency band of 0-18 Hz and completely overlaps with the artifacts, calculating AMSA directly from the corrupted VF signal will lead to an overestimation of its value (Coult et al., 2019). Using the reference waveform recorded from an additional channel independent of the ECG waveform but related to CPR artifacts, the artifacts can be modeled and suppressed by adaptive filters (Irusta et al., 2009; Ruiz de Gauna et al., 2014; Gong et al., 2017). However, the accuracy of shockable rhythm classification is still lower than that used for uncorrupted ECG waveforms. The primary reason for this result is that the artifacts are variable, and their characteristics depend on how the CC is administered and on the characteristics of the patient and the recording system (Ruiz de Gauna et al., 2014).

Recently, strategies have shifted from suppressing artifacts to mining hidden features with existing deep neural networks. Research was first performed in the ECG waveform without CPR. Picon et al. (2019) introduced an approach that combines a 2-layer CNN and a long short-term memory (LSTM) network for the detection of VF. Krasteva et al. (2020) demonstrated that the optimal hyperparameters of CNN with five convolutional blocks, a filter number between 5 and 20, and a kernel size of 10 resulted in maximum validation performance for the detection of shockable rhythm. Panda et al. (2020) used the first five decomposed subband signals, while Sabut et al. (2021) used a feature set of 24 time-frequency-based parameters of ECG waveforms as the input of CNN for shockable rhythm detection. Additionally, Ivanović et al. (2020) proposed a 3-stage CNN feature extractor model to predict defibrillation success. Using the raw 4 s VF signals immediately prior to the first shock in 260 adult patients, the strategy was demonstrated to be capable of learning useful representations for defibrillation outcome. These studies revealed that CNN approaches have equivalent or superior performance to classical feature extraction based machine learning algorithms and have the potential to be used in AEDs. Then, studies focused on rhythm analysis for the CC-corrupted ECG waveforms. Isasi et al. (2020) designed an algorithm to classify shockable rhythms using filtered ECG as the input of a CNN that contained 3 convolution blocks and 2 fully connected layers. Hajeb-M et al. (2021) designed a CNN model comprised of convolutional layers, residual networks, and bidirectional LSTM using 2-dimensional images that combined the ECG waveform with the amplitude and phase information derived from the short-time Fourier transform as input. Jekova and Krasteva (2021) showed that the optimal architecture of a deep CNN with the best performance for shockable rhythm analysis during CPR was with three convolutional layers when the raw ECG waveform was used. Although the datasets and validation methods of these studies were considerably different, their results unanimously confirmed that CNN can be used for reliable rhythm analysis during CPR.

In this study, we proposed an algorithm to estimate AMSA and guide CPR continuously during resuscitation effort using a 1D CNN. Instead of suppressing the CC-related artifacts and computing AMSA from the frequency domain, we implemented an end-to-end architecture of CNN, applying differentiated VF at the input and obtaining a continuous variable AMSA at the output, without determining the presence of CC with additional sensors. The architecture design and a high-quality database are the two primary determinants of the performance of CNN based methods. In this study, an architecture consisting of a 6-layer 1D CNN and three fully connected layers was developed based on previous studies concerning ECG waveform denoising, VF detection and defibrillation success prediction using CNN. Each block was composed of convolution, ReLU activation, dropout and max-pooling. Considering that the output result is a continuous variable rather than a classified variable, the classifier was composed of three fully connected perceptrons. The optimized hyperparameters of the model were similar to the results reported in previous studies, which further confirmed the robustness of the CNN network for VF signal feature extraction (Picon et al., 2019; Isasi et al., 2020; Krasteva et al., 2020; Panda et al., 2020; Hajeb-M et al., 2021; Jekova and Krasteva, 2021; Sabut et al., 2021). Conversely, the model must be trained by a representative and accurately labeled database with a sufficient sample size. Because it is impossible to have the underlying clean VF signals during CPR, prior efforts used simulated data that were constructed by randomly adding different scaled pure CC artifacts to the uncorrupted VF signals (Lo et al., 2013). Considering that VF is a quasi stationary signal, we used the adjacent uncorrupted signals as surrogate data of the corrupted VF to calculate AMSA. The simulated data indicated that the AMSA values between the uncorrupted segment pairs were highly correlated and that the AMSA value calculated from the uncorrupted VF could be used as the true value of the adjacent corrupted VF. The real-life dataset of corrupted/uncorrupted segment pairs was used to train the model due to the heterogeneity of CPR artifacts in real-life data and homogeneity of CPR artifacts in simulated data. As shown in Table 4, the performance of the proposed method is demonstrated to be superior to that of the traditional AMSA calculation method using simulated data and independent real-life CPR data (Zuo et al., 2021; Coult et al., 2022). Experimental results demonstrated the excellent feature extraction capability to exploit all information reflecting the energy state of the myocardium hidden in the VF signal. To our knowledge, this is the first study that uses a deep neural network model to calculate AMSA during uninterrupted CPR. This study indicated that the paradigm shift represented by end-to-end deep learning may enable a new approach to monitor the effectiveness of CC and predict defibrillation success during CPR using VF signals alone.

**TABLE 4:** Reported performance for assessment of Ventricular fibrillation prognosis in literatures.

Study	Method	Dataset	Achieved performance
Coult et al. (2019)	VF waveform was filtered using a bandpass filter of 1–30 Hz	Real-life data from 1,151 cardiac arrest patients	AUC was 0.75 without chest compression, and was 0.72 with chest compression
Lo et al. (2013)	CPR artifacts were removed using empirical mode decomposition and least square mean based adaptive filter	Simulated data from 150 VF and asystole patients with SNR of −9, −6, −3 and 0 dB	The limits of agreements were −1.11 to 1.49, −1.62 to 2.74, −1.64 to 4.36 and −3.11 to 9.77 for different levels of SNR. The AUC was 0.642 for original VF.
Coult et al. (2021)	CPR artifacts were filtered with variable-frequency notch filter. Ten ECG features and three dichotomous patient characteristics were combined usingsupport vector machines and logistic regression to predict outcome	Pre-shock data from 1,151 cardiac arrest patients	AUC for predicting defibrillation success was 0.74 during CPR and 0.77 without CPR. AUC for predicting functional survival was 0.75 during CPR and 0.76 without CPR.
Zuo et al. (2021)	CPR artifacts were filtered with a signal quality index and least mean square filter based adaptive filter	Pre-shock data from 512 cardiac arrest patients	AUC was 0.734 without chest compression, and was 0.713 with chest compression
Coult et al. (2022)	CPR artifacts were filtered with variable-frequency notch filter. Ten ECG features and three dichotomous patient characteristics were combined usingsupport vector machines and logistic regression to predict outcome	Real-life data from 434 cardiac arrest patients	AUC between 0.73–0.75 during resuscitation

There are several limitations/drawbacks that must be addressed regarding the current study. First, the dataset used to train the model was not uniformly distributed. As the samples with low AMSA values were much more abundant than those with high AMSA values of the real-life patient data, it is still unknown whether the overall performance of the proposed method will be improved and bias in the Bland-Altman plots will be decreased if a sufficiently large uniformly distributed dataset is available. Second, although the proposed method can provide continuous information about the dynamic changes in the AMSA during CPR, the VF features in their hidden layers were extracted blindly without a comprehensive explanation of the mechanism for future clinical application. Third, the model was established based on CC-corrupted VF signals using the least mean square method, so for the uncorrupted VF signals, such as segments during CC pauses and prior to shock delivery, the AMSA values would be underestimated. Four, the algorithm is designed to VF waveform analysis; thus, it cannot be used without the help of a reliable shockable rhythm detection algorithm. Although several algorithms have been demonstrated to correctly classify shockable rhythms during CPR using CNN, the performance of combining the current algorithm with those shockable rhythm detection algorithms also must be validated in future studies.

## 6 Conclusion

This study introduces a 1D CNN based continuous AMSA estimation approach, which is promising for reliable AMSA estimation during uninterrupted CPR using solely the ECG waveform. Experimental results indicate that this method performs better than traditional adaptive filtering and FFT-based techniques. Combined with the shockable rhythm detection algorithms during CC, the algorithm has the potential to be incorporated into current versions of AEDs, and personalized CPR can be implemented by real-time monitoring of the effectiveness of CC and predicting the probability of defibrillation success.

## Data Availability

The raw data supporting the conclusion of this article will be made available by the authors, without undue reservation.
